# Supplementary material for: A cross-country analysis of feasible income equality using the sigmoid function and the Boltzmann distribution
Source: PLoS One. 2025 Aug 8;20(8):e0329633. doi: 10.1371/journal.pone.0329633 (PMC12334062; doi:10.1371/journal.pone.0329633)
Supplement: S1 Table — (PDF) [file pone.0329633.s001.pdf]

Supporting Information for

A cross-country analysis of feasible income equality using the sigmoid function and the Boltzmann distribution

Thitithep Sitthiyot[1*], Kanyarat Holasut[2]

[1] Department of Banking and Finance, Faculty of Commerce and Accountancy, Chulalongkorn University, Bangkok, Thailand

[2] Department of Chemical Engineering, Faculty of Engineering, Khon Kaen University, Khon Kaen, Thailand

[*] Corresponding author

E-mail: thitithep@cbs.chula.ac.th (TS)

S1 Table. The calculated values of L, H, μ, and α of 71 countries.

Country	L	H	μ	α
Argentina	12.350	35.150	23.750	0.263
Armenia	15.550	29.750	22.650	0.423
Austria	15.500	30.650	23.075	0.396
Belgium	16.150	29.200	22.675	0.460
Benin	14.000	32.200	23.100	0.330
Burkina Faso	13.150	33.200	23.175	0.299
Bulgaria	13.250	33.750	23.500	0.293
Bolivia	12.850	34.550	23.700	0.276
Brazil	9.800	38.550	24.175	0.209
Central African Republic	11.900	35.300	23.600	0.256
China	13.550	32.750	23.150	0.313
Cote d'Ivoire	13.650	32.550	23.100	0.317
Cameroon	12.150	35.200	23.675	0.260
Colombia	9.150	39.300	24.225	0.199
Costa Rica	10.500	37.350	23.925	0.223
Cyprus	14.800	30.950	22.875	0.372
Czechia	15.950	29.150	22.550	0.455
Denmark	15.600	29.800	22.700	0.423
Dominican Republic	13.150	33.600	23.375	0.293
Ecuador	11.450	36.250	23.850	0.242
Spain	14.750	32.000	23.375	0.348
Estonia	14.500	31.500	23.000	0.353
Finland	15.600	29.700	22.650	0.426
France	15.250	30.950	23.100	0.382
United Kingdom	14.800	31.350	23.075	0.363
Georgia	14.500	32.000	23.250	0.343
Guinea-Bissau	14.050	32.000	23.025	0.334
Greece	14.850	31.600	23.225	0.358
Croatia	15.750	30.150	22.950	0.417
Hungary	15.450	30.050	22.750	0.411
Indonesia	13.550	32.600	23.075	0.315
India	14.300	31.750	23.025	0.344
Ireland	15.050	30.550	22.800	0.387
Iran, Islamic Rep.	13.900	32.650	23.275	0.320
Israel	13.850	33.400	23.625	0.307
Italy	14.500	32.200	23.350	0.339

(Continued)

S1 Table. The calculated values of L, H, μ, and α of 71 countries.

Country	L	H	μ	α
Jamaica	12.750	34.400	23.575	0.277
Kazakhstan	14.700	30.400	22.550	0.382
Kenya	12.800	33.600	23.200	0.288
Kyrgyz Republic	15.050	30.200	22.625	0.396
Korea, Rep.	14.650	31.600	23.125	0.354
Lithuania	13.650	32.850	23.250	0.313
Luxembourg	14.800	31.550	23.175	0.358
Latvia	14.350	32.100	23.225	0.338
Moldova	15.900	29.050	22.475	0.456
Mali	13.450	32.750	23.100	0.311
Montenegro	14.650	32.200	23.425	0.342
Malaysia	12.600	34.500	23.550	0.274
Niger	14.150	31.500	22.825	0.346
Netherlands	16.450	28.850	22.650	0.484
Panama	10.200	37.900	24.050	0.217
Peru	12.900	34.200	23.550	0.282
Philippines	12.350	34.400	23.375	0.272
Poland	15.750	29.950	22.850	0.423
Portugal	14.150	32.150	23.150	0.333
Paraguay	12.050	35.200	23.625	0.259
Romania	15.050	31.950	23.500	0.355
Russian Federation	14.000	32.550	23.275	0.323
Senegal	13.550	32.750	23.150	0.313
El Salvador	13.400	33.850	23.625	0.293
Serbia	15.000	31.450	23.225	0.365
Slovak Republic	17.100	28.350	22.725	0.533
Slovenia	16.400	28.500	22.450	0.496
Sweden	15.650	30.450	23.050	0.405
Togo	13.200	33.400	23.300	0.297
Thailand	13.600	32.600	23.100	0.316
Tonga	15.700	29.650	22.675	0.430
Tunisia	14.350	31.750	23.050	0.345
Turkiye	11.850	35.550	23.700	0.253
Uruguay	12.650	34.500	23.575	0.275
United States	13.000	33.950	23.475	0.286
