# Supplementary material for: A cross-country analysis of feasible income equality using the sigmoid function and the Boltzmann distribution
Source: PLoS One. 2025 Aug 8;20(8):e0329633. doi: 10.1371/journal.pone.0329633 (PMC12334062; doi:10.1371/journal.pone.0329633)
Supplement: Attachment — Submitted filename: Response to Reviewers_R2.pdf [file pone.0329633.s004.pdf]

Response to Reviewers

Manuscript Number: PONE-D-25-15291R2

Title: A cross-country analysis of feasible income equality using the sigmoid function and the Boltzmann distribution

We sincerely thank Reviewer #1 and Reviewer #2 for recommending our revised manuscript for publication in *PLOS ONE* on the condition that minor adjustments are made. We would like to inform both Reviewers that we have revised our manuscript according to suggestions made by both Reviewers. They are as follows.

Reviewer #1:

The authors have appropriately addressed major concerns raised in the initial round of review. I find the revised manuscript improved and recommend acceptance after minor polishing.

The following sentence in the manuscript reads somewhat awkwardly due to its length and structure:

Given that the sigmoid function is used in well-being and welfare analysis, for example, Hänsel and Quaas [27], Takano et al. [28], and Mancini and Vecchi [29] and the Boltzmann distribution is employed to study income and wealth distributions, for example, Drăgulescu and Yakovenko [30], Pal and Pal [31], Pareschi and Toscani [32], Tao [33], and Ludwig and Yakovenko [34], Park and Kim [26] propose that the sigmoid function can be used jointly with the Boltzmann distribution in order to calculate the optimal income distribution that represents feasible income equality and maximizes total social welfare simultaneously.

I suggest revising it for clarity and natural flow. One possible revision is:

Given that the sigmoid function has been used in well-being and welfare analysis (e.g., Hänsel and Quaas [27]; Takano et al. [28]; Mancini and Vecchi [29]) and that the Boltzmann distribution has been applied to the study of income and wealth distributions (e.g., Drăgulescu and Yakovenko [30]; Pal and Pal [31]; Pareschi and Toscani [32]; Tao [33]; Ludwig and Yakovenko [34]), Park and Kim [26] propose using both the sigmoid function and the Boltzmann distribution to calculate the optimal income distribution that represents feasible income equality and maximizes total social welfare.

We thank Reviewer #1 for this suggestion. We adjust the sentence that Reviewer #1 kindly rewrites it for us so that it is in line with *PLOS ONE*'s citation style in Introduction, paragraph 2 in our revised manuscript. It reads as follows.

Given that the sigmoid function has been used in well-being and welfare analysis [27-29] and that the Boltzmann distribution has been applied to the study of income and wealth distributions [30-34], Park and Kim [26] propose using both the sigmoid function and the Boltzmann distribution to calculate the optimal income distribution that represents feasible income equality and maximizes total social welfare.

Reviewer #2:

The manuscript provides robust empirical evidence that the gap between actual and optimal income distributions across 71 countries is closely related to institutional quality, especially the rule of law. The authors also present a clear functional relationship—using a sigmoid function—that enables the estimation of feasible income shares from actual income shares, offering a practical quantitative benchmark for policy design. Policy implications and real-world limitations are discussed in a balanced and convincing manner. Furthermore, the authors have responded diligently and thoroughly to all reviewer comments.

For minor revision, I recommend that the authors simply clarify in the Abstract that "feasible income equality" refers to the 'optimal income distribution that is realistically attainable.' No further substantial changes are required.

Overall, I find the manuscript to be of high quality and suitable for publication after this minor clarification.

We thank Reviewer #2 for this useful recommendation. We add the sentence that Reviewer #2 suggests in the Abstract in our revised manuscript.
